# Methotrexate-induced epidermal necrosis following intrathecal chemotherapy: A case report

**DOI:** 10.1016/j.jdcr.2024.12.005

**Published:** 2024-12-14

**Authors:** Brad R. Woodie, Stephany L. Vittitow, Sweta Subhadarshani

**Affiliations:** a Department of Dermatology, University of Cincinnati College of Medicine, Cincinnati, Ohio; b Department of Dermatology, University of Pennsylvania, Perelman School of Medicine, Philadelphia, Pennsylvania

**Keywords:** drug eruption, leukemia, methotrexate-induced epidermal necrosis, oncodermatology, oncology, toxic erythema of chemotherapy

## Introduction

Methotrexate-induced epidermal necrosis (MEN) is a rare but potentially life-threatening condition that presents as widespread skin necrosis.^1^^,^^2^ Older patients and those with chronic kidney disease are at an elevated risk for MEN due to decreased renal clearance of methotrexate.^1^^,^^2^ The clinical presentation of MEN may overlap with subtypes of toxic erythema of chemotherapy (TEC), a more common adverse reaction that can also be caused by methotrexate.^3^ Distinguishing MEN from TEC is important due to differing management and prognostic implications, with MEN having a mortality rate of 10% to 30%.^1^, ^2^, ^3^ This case report presents a patient whose cutaneous reaction to intrathecal methotrexate was initially believed to be TEC, but upon further review, MEN is considered more likely.

## Case report

**Fig 1:**
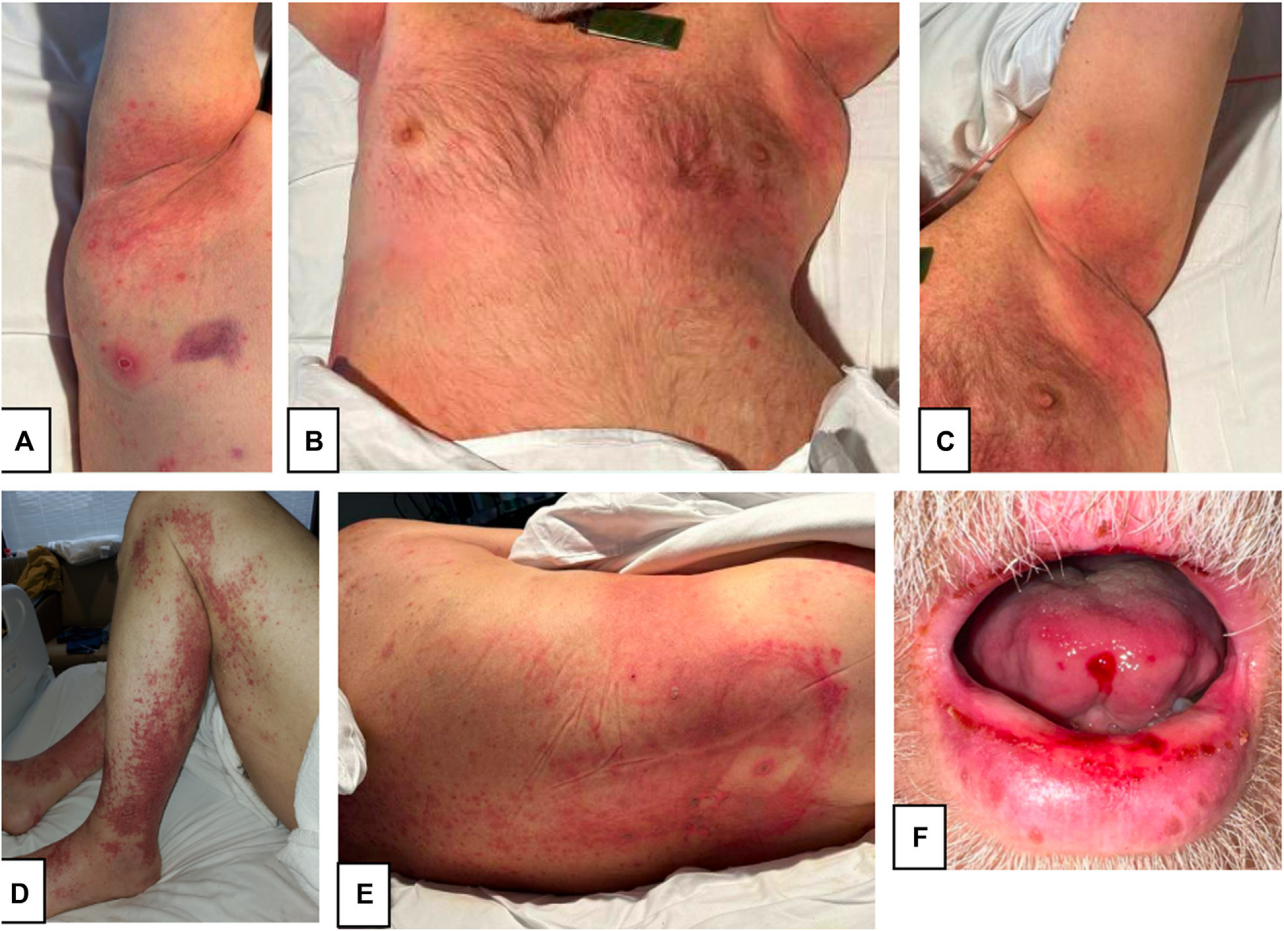
Methotrexate-induced epidermal necrosis at time of initial presentation. (**A** and **C**): Thin erythematous patches and plaques in axillae, chest (**B**) and legs (**D**). **E,**: Involvement of back with full thickness skin necrosis. **F,**: Erosions of the lips and tongue.

A 60-year-old male with a history of acute myeloid leukemia undergoing intravenous chemotherapy with 5-azacitidine and venetoclax for 10 months was admitted with new weakness and altered mental status. Further evaluation revealed acute disseminated encephalomyelitis due to central nervous system involvement of acute myeloid leukemia, and intrathecal chemotherapy was initiated. He received cytarabine 70 mg, methotrexate 12 mg, and hydrocortisone 50 mg intrathecally on hospital days 7 and 10. On hospital day 13, the patient developed a diffuse, painful cutaneous eruption affecting the body and oral mucosa accompanied by a fever of 101.3 °F. Physical examination was notable for several moderately well-defined, pink patches, and thin plaques involving the bilateral axillae, groin, inguinal folds, and bilateral lower extremities (Fig 1). There were scattered deroofed and tense bullae of the scrotum, flanks, and back, and there were areas of full thickness skin necrosis and desquamation on the back. There was an erosion of the tip of the tongue and hemorrhagic crust overlying the vermilion lips. Diaphoresis was noted as well. Laboratory examination was notable for pancytopenia with a white blood cell count of 0.9 × 10^3^ μL/mL, hemoglobin of 6.7 g/dL, and platelets of 35 × 10^3^ μL/mL. Renal function panel was notable for creatinine of 3.4 mg/dL. Differential diagnosis at initial dermatologic consultation included TEC, Stevens-Johnson syndrome/toxic epidermal necrolysis, and less likely leukemic involvement of the skin.

**Fig 2:**
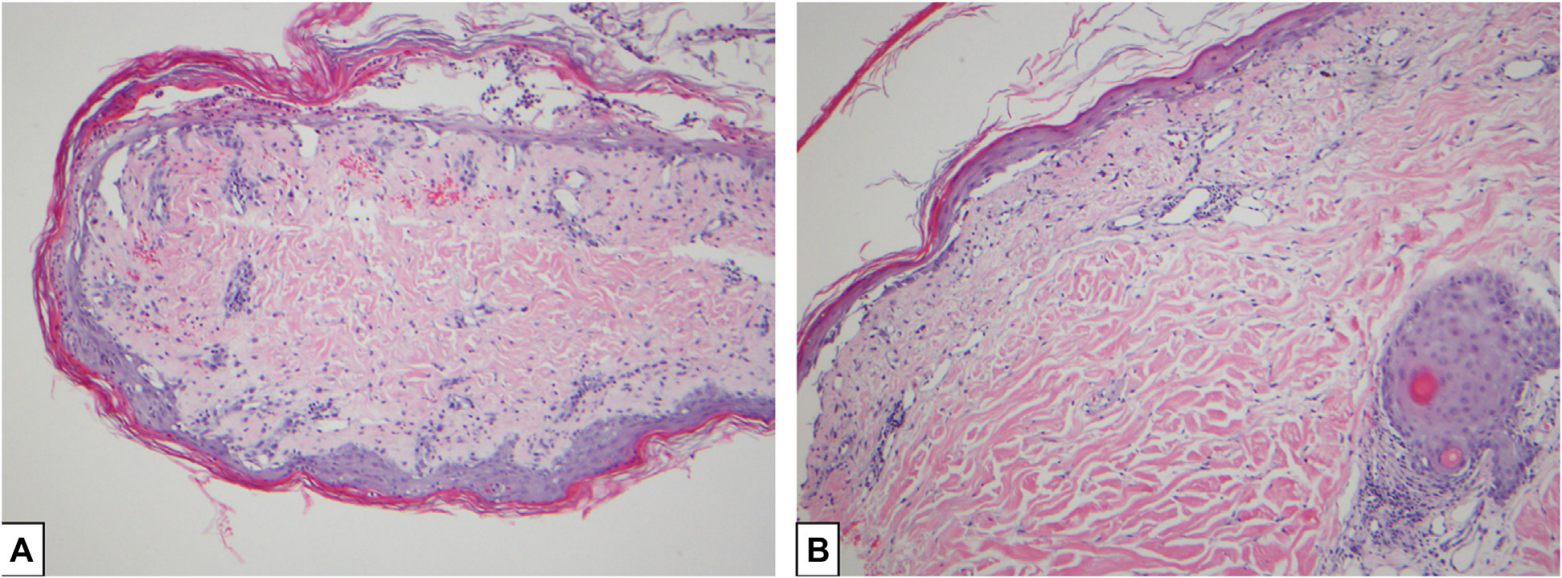
Methotrexate-induced epidermal necrosis skin biopsies (hematoxylin and eosin) of the right flank (100×) (**A**) and right shoulder (200×) (**B**). Vacuolar interface dermatitis with subepidermal clefting, epidermal atrophy, and a scant superficial perivascular lymphocytic infiltrate were seen. No findings of eccrine squamous syringometaplasia or epidermal dysmaturation.

Skin biopsies showed severe vacuolar interface dermatitis, epidermal atrophy, and subepidermal clefting (Fig 2).

**Fig 3:**
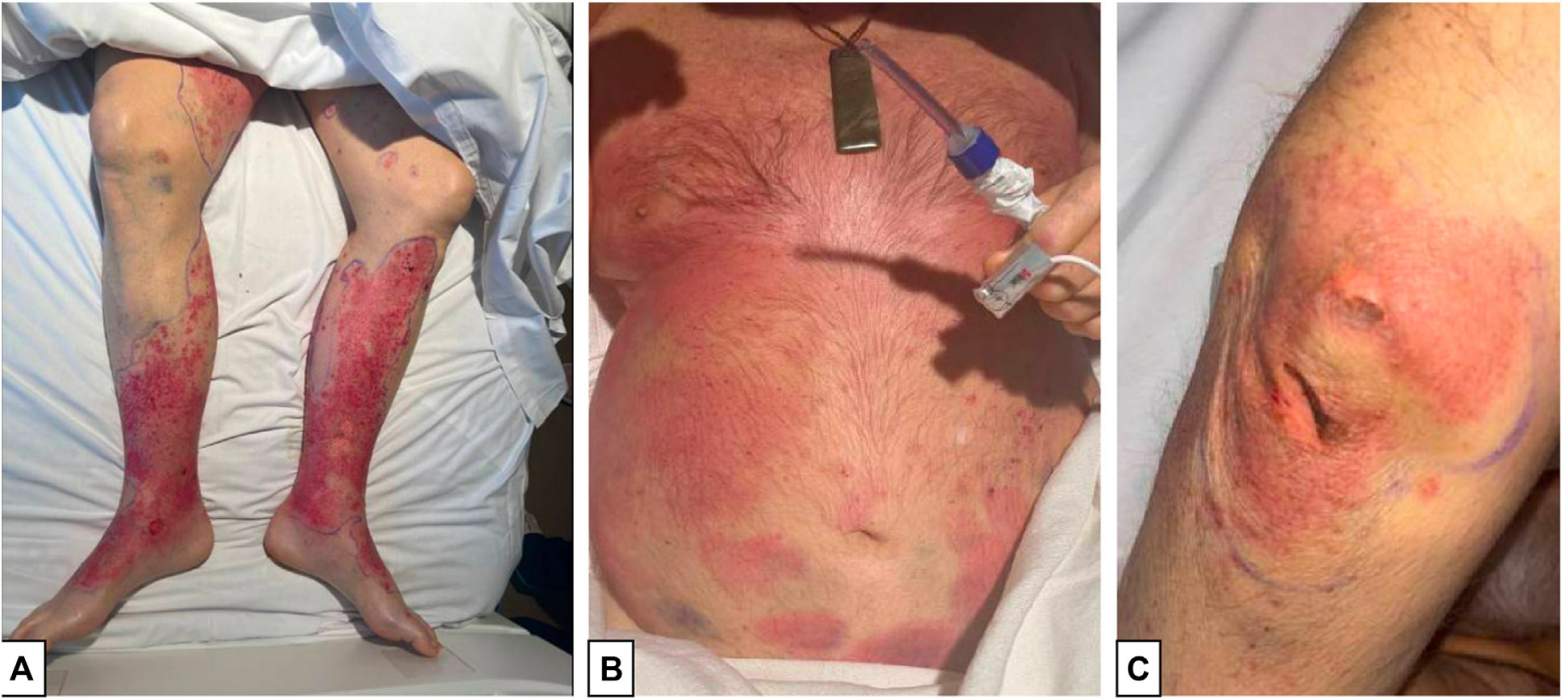
Progression of methotrexate-induced epidermal necrosis 3 days after presentation and cessation of chemotherapy. **A,**: Legs showing distal focal areas of necrosis within the erythematous plaques. **B,**: The plaques involving the trunk progressed to a dusky red. **C,**: New desquamation of the olecranal skin.

Topical clobetasol 0.05% cream was begun twice daily in addition to white petrolatum and nonadherent dressings. Chemotherapy was discontinued; however, the patient’s lesions continued to worsen with progression of skin sloughing (Fig 3). Treatment with intravenous immunoglobulin was deferred due to the risk of pulmonary edema in the setting of declining kidney function. The patient transitioned to comfort care on hospital day 20 and passed away on hospital day 22.

## Discussion

The patient’s skin necrosis following initiation of intrathecal methotrexate and cytarabine was initially attributed to TEC. After further review, we believe that MEN is the more likely diagnosis. Intrathecal methotrexate is favored as the inciting agent because intrathecal cytarabine (a common cause of TEC) is rapidly eliminated by hepatic cytidine deaminase after resorption from cerebrospinal fluid.^4^ While the intrathecal route of administration is thought to reduce systemic drug toxicity, intrathecal methotrexate can still reach clinically significant systemic concentrations.^5^^,^^6^ Methotrexate is well-known to cause dose-dependent mucositis and pancytopenia,^7^ but cutaneous ulceration and necrosis is rare. The entity now termed “methotrexate-induced epidermal necrosis” was first recognized as cutaneous ulcers appearing in patients receiving low-dose methotrexate for psoriasis.^2^ More recent reports describe MEN in patients receiving methotrexate for nonpsoriatic conditions or malignancy.^1^^,^^2^^,^^8^

Although MEN can share overlapping clinical features with subtypes of TEC, several details in this case align more closely with MEN. Classic TEC histological findings, such as eccrine squamous syringometaplasia and epidermal dysmaturation^2^^,^^9^ were notably absent. Biopsies instead showed epidermal atrophy, vacuolar interface dermatitis, and a mild lymphocytic infiltrate, which are all characteristic of MEN.^1^^,^^2^ The involvement of intertriginous areas is typical for the malignant intertrigo subtype of TEC,^10^ but similar patterns are also seen in MEN.^2^ The current patient also exhibited markedly impaired renal function and pancytopenia, both of which are key risk factors for MEN.^1^^,^^2^ Moreover, MEN is more likely to occur in patients receiving high doses of methotrexate without folic acid supplementation, as is common in IT regimens used for central nervous system malignancies.^1^^,^^2^ Serum methotrexate was not assessed in the current case, but a dose-response relationship between serum levels of methotrexate and MEN has been suggested.^1^^,^^2^

Prognosis and optimal management strategies differ between MEN and TEC. As both conditions result from direct toxicity to keratinocytes, methotrexate should be stopped when a severe reaction is noted. Leucovorin rescue therapy has a theoretical benefit in patients with MEN via reducing further methotrexate-induced cell death.^1^^,^^7^ Chen et al (2017) did not find a significant difference in mortality or recovery time between patients who received leucovorin and patients who did not, but their case series was limited by a small sample size.^1^ Some patients in their case series were given systemic corticosteroids or granulocyte colony-stimulating factor, but the mortality or recovery time benefits are unclear.^1^

This case underscores the importance of considering MEN as a cause of skin necrosis after administration of methotrexate, even intrathecally. Diagnosing MEN is challenging due to the rarity of the condition. Additional reports are needed to better characterize MEN and determine optimal management strategies.

## Conflicts of interest

None disclosed.

## References

[1] Chen T.J., Chung W.H., Chen C.B. (2017). Methotrexate-induced epidermal necrosis: a case series of 24 patients. J Am Acad Dermatol.

[2] Berna R., Rosenbach M., Margolis D.J., Mitra N., Baumrin E. (2022). Methotrexate cutaneous ulceration: a systematic review of cases. Am J Clin Dermatol.

[3] Bolognia J.L., Cooper D.L., Glusac E.J. (2008). Toxic erythema of chemotherapy: a useful clinical term. J Am Acad Dermatol.

[4] Slevin M.L., Piall E.M., Aherne G.W., Harvey V.J., Johnston A., Lister T.A. (1983). Effect of dose and schedule on pharmacokinetics of high-dose cytosine arabinoside in plasma and cerebrospinal fluid. J Clin Oncol.

[5] Bostrom B.C., Erdmann G.R., Kamen B.A. (2003). Systemic methotrexate exposure is greater after intrathecal than after oral administration. J Pediatr Hematol Oncol.

[6] Singhal R., Daily K., Wheeler S. (2023). Systemic absorption of intrathecal methotrexate. BMJ Case Rep CP.

[7] Howard S.C., McCormick J., Pui C.H., Buddington R.K., Harvey R.D. (2016). Preventing and managing toxicities of high-dose methotrexate. Oncol.

[8] Lewis H.A., Nemer K.M., Chibnall R.J., Musiek A.C. (2017). Methotrexate-induced cutaneous ulceration in 3 nonpsoriatic patients: report of a rare side effect. JAAD Case Rep.

[9] Martorell-Calatayud A., Sanmartín O., Botella-Estrada R. (2011). Chemotherapy-related bilateral dermatitis associated with eccrine squamous syringometaplasia: reappraisal of epidemiological, clinical, and pathological features. J Am Acad Dermatol.

[10] Smith S.M., Milam P.B., Fabbro S.K., Gru A.A., Kaffenberger B.H. (2016). Malignant intertrigo: a subset of toxic erythema of chemotherapy requiring recognition. JAAD Case Rep.

